# Cognitive Behavioral Therapy Alone or in Combination with Eszopiclone in Comorbid Insomnia and Obstructive Sleep Apnea in Veterans with Posttraumatic Stress Disorder: A Randomized Trial

**DOI:** 10.1007/s11126-025-10143-9

**Published:** 2025-04-09

**Authors:** Ali A. El-Solh, Yolanda Lawson, Amber Martinson, Gregory Wilding

**Affiliations:** 1 VA Western New York Healthcare System, Research and Development, Buffalo, NY USA; 2 Division of Pulmonary, Critical Care, and Sleep Medicine, Department of Medicine, Jacobs School of Medicine, Buffalo, NY USA; 3 Department of Epidemiology and Environmental Health, School of Public Health and Health Professions, Buffalo, NY USA; 4 Department of Biostatistics, School of Public Health and Health Professions, University at Buffalo, Buffalo, NY USA; 5 Behavioral Health Service, George Wahlen VA Medical Center, Salt Lake City, UT USA; 6 Western New York Healthcare System, 3495 Bailey Avenue, Buffalo, NY 14215 USA

**Keywords:** Cognitive behavioral therapy, Insomnia, Sleep apnea, Pharmacotherapy, Continuous positive airway pressure

## Abstract

We sought to assess the augmentation of brief behavioral therapy for insomnia (BBTI) with eszopiclone (ESZ) over BBTI alone for the treatment of chronic insomnia on sleep quality, sleep indices, and continuous positive airway pressure (CPAP) adherence in PTSD veterans with COMISA. The pilot trial involved 53 PTSD patients (46 males and 7 females, mean age 48.2±8.3 years) with COMISA randomized to combination therapy of BBTI plus 2 weeks of eszopiclone (2 mg/d) or BBTI alone with follow-up visits conducted at 6 and 24 weeks. The main outcome measure was sleep quality assessed by the Pittsburgh Sleep Quality Index (PSQI). A significant decrease in PSQI scores was observed between baseline and 24 weeks for BBTI plus ESZ (-5.24 [95% CI, -6.55 to -3.94]; *p* < 0.001) and BBTI-only (-5.45 [95%CI, -6.75 to -4.14]; *p* < 0.001). No significant group allocation effects x time interactions were detected. Similar improvements were recorded for ISI between baseline and 24 weeks (BBTI plus ESZ (-8.32 [95%CI, -10.51 to -6.14]; *p* < 0.001) and BBTI-only (-8.64 [95%CI, -10.88 to -6.41]; *p* < 0.001)) with no interaction effect between treatment groups x time. Combination therapy produced a higher remission rate of insomnia at 6 weeks, with both interventions achieving comparable rates at 24 weeks. Results of the mixed effect models for CPAP use revealed no group x time interaction effects. In patients with COMISA, the combination of eszopiclone with BBTI resulted in comparable improvement in sleep quality of life to that achieved with BBTI-only therapy. Although the addition of eszopiclone to BBTI conferred an early benefit in remission rate of insomnia relative to BBTI, both modalities achieved similar outcomes at long-term follow-up.

**Clinical Trial Registration** This study was registered with ClinicalTrials.gov (Identifier NCT03937713).

## Introduction

Among the myriads of sleep disorders encountered in Veterans with PTSD, the coexistence of comorbid insomnia and OSA, a condition originally termed “COMISA” [1], is identified in 38.2% of patients presenting for initial evaluation of sleep disturbances [2, 3]. Consistent with other studies [4, 5], veterans with COMISA experience greater quality of life impairments, more severe manifestations of PTSD, and more frequent psychiatric symptoms than those with either disorder alone [6, 7]. Traditionally, treatment models have focused on targeting one disorder at a time [8, 9]. The decision is usually guided by which disease is diagnosed first or by which constellation of symptoms is manifested on presentation. Clinical investigations have elucidated a distinct cluster of patients characterized by frequent nocturnal awakenings and elevated apnea-hypopnea index (AHI), whereby continuous positive airway pressure (CPAP)—the only treatment—may alleviate both insomnia and OSA symptoms [10, 11]. However, in most cases, the presence of insomnia contributes to poor CPAP adherence [12, 13], leaving both sleep conditions untreated.

Given that cognitive behavior therapy for insomnia (CBT-I) is considered the preferred primary treatment for insomnia [14], recent clinical investigations have explored the benefits of incorporating CBT-I concomitantly with or prior to introducing CPAP therapy [15]. Although four randomized controlled trials (RCTs) concluded that CBT-I improves insomnia symptoms in patients with COMISA and can be safely administered prior to OSA treatment [16–19],, two RCTs found no significant benefit of CBT-I for improving CPAP adherence [17, 18]. 

An alternative model is to augment CBT-I with pharmacotherapy to attenuate the state of hyperarousal while patients are assimilating their self-management skills. This combination therapy has been tested in several RCTs in patients with *insomnia only* [20–23]. When compared to CBT-I, combination therapy produced substantial advantages in the trajectory of sleep onset latency, sleep efficiency, wake after sleep onset, and sleep quality during the first week of treatment, and displayed a sustained response in total sleep time over a few weeks [24]. More importantly, patients who received time-limited pharmacotherapy did better than those who continued using medication intermittently [20, 25]. A favorable outcome was best observed when pharmacotherapy was initiated during CBT-I delivery and tapered prior to CBT-I completion [26]. This therapeutic approach has not been tested in veterans with COMISA. The objectives of this pilot study were to evaluate the comparative effectiveness of brief behavioral therapy for insomnia (BBTI) plus eszopiclone, a non-benzodiazepine ϒ-aminobutyric acid-A receptor agonist, versus BBTI-only on sleep quality in veterans with PTSD experiencing COMISA and to determine the treatment effects of combination therapy versus BBTI-only on insomnia severity, sleep propensity, depression, burden of PTSD, and CPAP utilization.

## Methods

### Design

This is a pilot, open-label, randomized comparator trial of combined BBTI with eszopiclone versus BBTI-only in veterans with PTSD presenting with COMISA. The study was approved by the Western New York Veterans Affairs Institutional Review Board. The study was planned and implemented in concordance with the Consolidated Standards of Reporting Trials (CONSORT) for randomized clinical trials [27]. The study was performed in accordance with the ethical standards as laid down in the 1964 Declaration of Helsinki.

### Participants

Participants were recruited through face-to-face contact or via targeted mail after initial pre-screening of the medical records for sleep apnea. Veterans between the ages of 18 and 65 years were considered for enrollment if: (1) they had PTSD as determined using the Diagnostic and Statistical Manual of Mental Disorders, fifth edition diagnostic criteria (DSM-5) criteria and based on a score of 25 or higher on the Clinician-Administered PTSD Scale for DSM-5 [28]; (2) they met the DSM-5 criteria for insomnia disorder [29], 2) had documented OSA by polysomnography (AHI ≥ 5 or more/hour), and (3) were non-adherent to CPAP as defined by device usage of < 4 h per night. Exclusion criteria included: (1) prior history of narcolepsy, restless leg syndrome, central sleep apnea or parasomnias, (2) unstable psychiatric condition (e.g., schizophrenia, active suicidal ideations), (3) ongoing substance use disorder, (4) bipolar disorder, (5) pregnancy, (6) dementia, (7) epilepsy, (8) shift work, (9) use of potent cytochrome p4503A4 inhibitor medications, (10) history of complex nocturnal behavior, 11) significant hepatic impairment, 12) history of hypersensitivity, intolerance to eszopiclone, and 13) prior behavioral or current pharmacological treatment for insomnia.

Participants who fulfilled the inclusion/exclusion criteria were evaluated for insomnia and depression using the Insomnia Severity Index (ISI) [30] and the Beck Depression Inventory (BDI-II) Questionnaire [31]. Participants who scored < 15 on the ISI, which is the recommended cutoff to detect clinical insomnia according to the American Academy of Sleep Medicine cases [32], or > 30 on the BDI-II questionnaire indicating severe depression were excluded from participation. After obtaining informed consent, baseline demographic, anthropomorphic, and socioeconomic data were collected. Additional information on comorbidities, coexisting psychiatric disorders, and medication use, including the last CPAP smartcard reading, was also recorded during that visit. Each participant was also asked to complete the Epworth Sleepiness Scale (ESS) [33], PTSD Checklist (PCL) [34], and the Pittsburgh Sleep Quality Index (PSQI) [35]. After collecting baseline information, all participants were asked to prospectively track their sleep/wake patterns using the Pittsburgh Sleep Diary [36] for the seven consecutive days and nights that immediately preceded their treatment visits. A wrist actigraph was assigned to all participants to monitor their sleep/wake schedule. Participants were asked to wear it for the seven consecutive days that immediately preceded the study intervention.

### Randomization

Participants who completed all baseline assessments were randomized in a 1:1 fashion to either BBTI plus eszopiclone or BBTI-only using a stratified permuted block randomization scheme with the single stratification variable(s) being sleep severity using AHI = 5 to AHI < 15 for mild OSA and AHI ≥ 15 for moderate to severe OSA.

### Interventions

BBTI was administered individually by trained staff therapists under the supervision of a licensed clinical psychologist as detailed in Online Resource. A concise checklist for the topics pertaining to each session was used to avoid drifting from the protocol contents. BBTI was adapted from a manualized behavioral treatment as described previously [37]. Patients assigned to BBTI plus eszopiclone received BBTI plus 2 mg of eszopiclone nightly, 30 min before bedtime for a period of 2 weeks. A brief in-person consultation was provided during the enrollment visit to review potential side effects that could arise from the use of eszopiclone.

### Follow-up Study Visits

Follow-up visits for all participants were scheduled at 6- and 24 weeks post-randomization. At each of the scheduled follow-up visits, participants were asked to complete the PSQI (primary outcome) [35], ESS [33], ISI [30], PCL [34], and BD-II [31]. Patients were considered *treatment responders* if their ISI change score compared with baseline was greater than 7 and *treatment remitters* if their absolute ISI score was less than 8 [38]. Actigraphy data was also retrieved at each of these time points.

The list of medications was reviewed and any changes in prescribed and non-prescribed medicines were recorded. During these follow-up visits, CPAP use was assessed by downloading the Smartcard data by a respiratory therapist who was blinded to the treatment intervention. CPAP utilization was derived from the average use across all nights, while CPAP adherence was obtained from the percentage of nights with CPAP use ≥ 4 h/night during the past 30 days.

### Outcome Measures

The primary outcome measure was the Pittsburgh Sleep Quality Index. Secondary outcomes consisted of ISI, ESS, BDI-II, PCL, and actigraphy-derived sleep indices. A more detailed description of outcome measures is posted on the online supplement.

### Statistical Analysis

The sample size selected for this preliminary trial was based on the analysis of the PSQI as the primary outcome. Calculations were approximated by those of a simple two-sample t-test for mean differences in PSQI in response to BBTI [39]. The estimate of the variability used for sample size calculations was derived from a randomized controlled trial of BBTI for the treatment of insomnia in military Veterans where an approximate standard deviation of 3.5 was documented [39]. Assuming a correlation coefficient of 0.7 between pre-and post-treatment [40], 21 patients per arm were needed to achieve 80% power at a significance level of 5% in order to detect a clinically significant difference of 3 units in PSQI [41]. To compensate for an anticipated attrition rate of 25%, the recruitment target was set at 52 participants.

All analyses were based on the intention-to-treat principle. The student *t*-test was applied for continuous variables and the chi-square test for categorical variables to test differences in baseline characteristics between the treatment groups. To examine group differences in outcome measures, we derived a linear mixed-effects model (LMM) using changes assessed at 6 weeks and 24 weeks in all randomized participants. The model involved two vectors: Vector 1 represented changes between baseline and week 6 and Vector 2 reflected changes between week 6 and week 24. Participants were included as random effect in the model. Group allocation (BBTI plus ESZ or BBTI-only) and the interaction with Vector 1 and Vector 2 were fitted as fixed effects while controlling for age, sex, AHI, and outcome value at baseline [42]. An unstructured variance-covariance matrix was specified between repeated measures on the same individual. With the assumption that all the data was missing at random, the maximum likelihood inference was considered unbiased. Actigraphy data were analyzed using the same method. The significance level (two-tailed) was set at p-values lower than 0.05. Alpha level was adjusted using the Bonferroni correction when indicated. All analyses were performed using STATA (v 16.0, StataCorp LP).

## Results

### Study Population

**Fig. 1:**
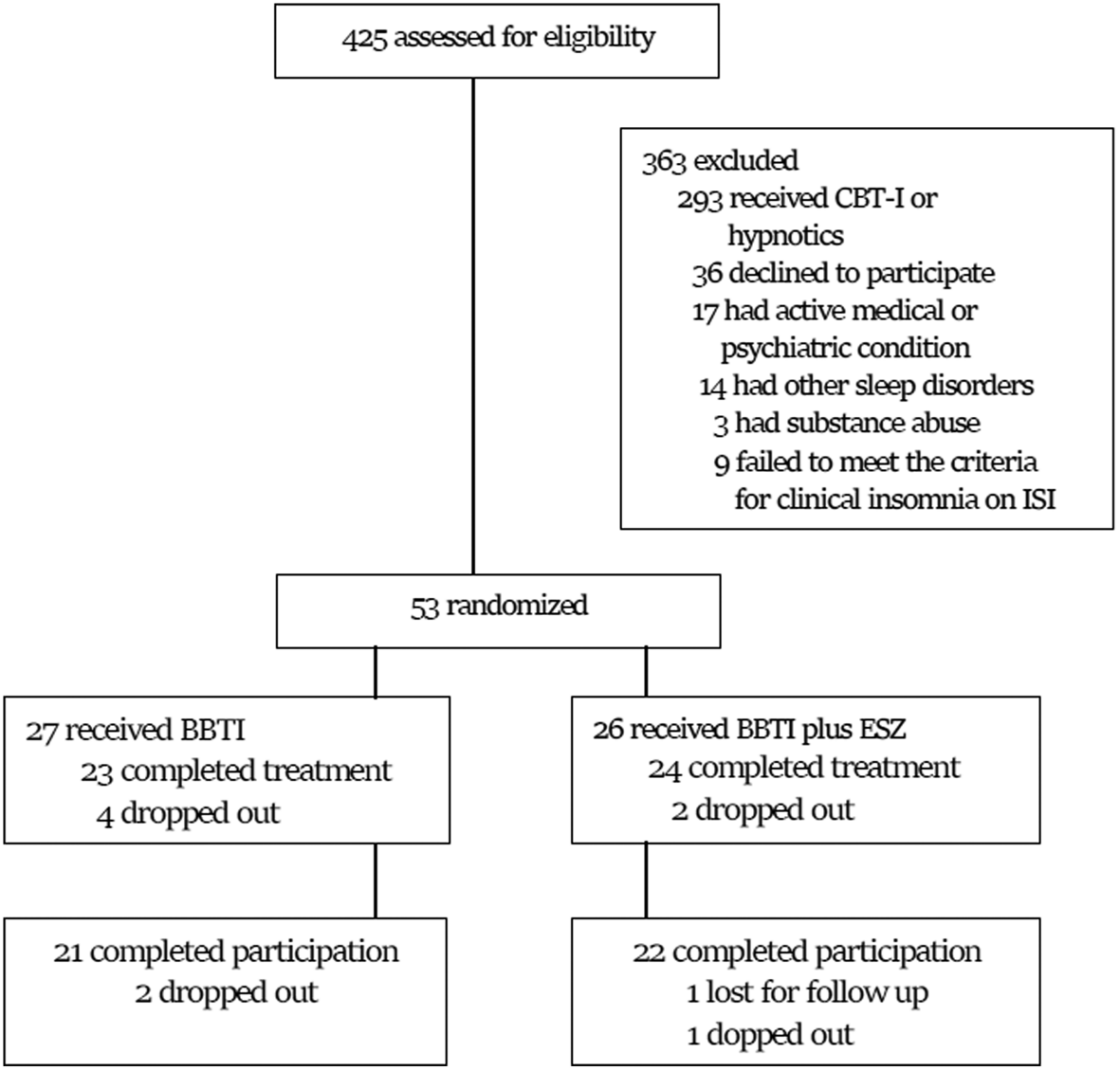
CONSORT patient flow chart

During the period extending from January 4th, 2020, and December 28th, 2023, we assessed 425 veterans with PTSD for eligibility. Figure 1 shows the flow diagram of enrollees throughout the study period. Three hundred sixty-three veterans were excluded because of prior treatment with CBT-I and/or active use of hypnotics (81%), presence of concurrent active medical or unstable psychiatric conditions (5%), active substance abuse (1%), documented sleep disorders other than COMISA (4%), or unable to commit for the duration of the trial (9%). Of the 62 veterans who met eligibility, nine did not meet the criteria for clinical insomnia by ISI. The remaining 53 veterans were randomized to BBTI plus ESZ (*n* = 26) or BBTI-only (*n* = 27). During the 6-week treatment phase, 23 completed all four sessions in the BBTI group compared to 24 in the BBTI plus ESZ. In total, ten participants (19%) did not complete all the scheduled visits. One was lost for follow up and nine withdrew, including six from the BBTI-only group and three from the BBTI plus ESZ. The attrition rate was not significantly different between treatment groups. The most common reasons for withdrawing were lack of benefit from intervention (*n* = 5), conflict with work schedule (*n* = 3), and personal circumstances (*n* = 1).

Baseline demographic and clinical characteristics of the study population are summarized in Table 1. The majority of participants (76%) reported both difficulty falling asleep and maintaining sleep. The mean insomnia duration was 9.7 (6.2) years. Although none of the patients were on pharmacotherapy for insomnia, 18 patients (34%) were exposed to at least one hypnotic/sedative prior to enrollment. Based on overnight polysomnography (PSG), 11% had mild, 21% had moderate, and 68% had severe OSA. All PSG-derived sleep indices (TST, SOL, AHI, or nadir oxygen saturation) were comparable between the BBTI-only group and the BBTI plus ESZ group at baseline. Audited recordings of BBTI sessions achieved a mean overall fidelity of 95.6% (95% CI 92.3–100.0), suggesting very good integrity. Compliance with eszopiclone regimen was 94% based on pill count.

**Table 1:** Baseline demographic and polysomnographic characteristics of the two treatment groups

	BBTI plus ESZ (*N* = 26)	BBTI (*N* = 27)	*P* value
Age, years	48.0 ± 7.3	48.4 ± 9.3	0.88
BMI, kg/m^2^	32.8 ± 6.8	33.0 ± 6.6	0.90
Sex, n (%)			0.65
Male	22 (84)	24 (89)	
Female	4 (16)	3 (11)	
**Race/ethnicity**, n (%)			0.28
Black or African American	4 (15)	8 (29)	
Caucasian/White	21 (81)	18 (71)	
Hispanic or Latino	1 (4)	0	
**Marital Status**, n (%)			0.44
Single/Never married	7 (27)	8 (29)	
Married/Living with domestic partner	14 (54)	17 (65)	
Divorce/Separated	5 (19)	2 (7)	
**Employment status**, n (%)			0.32
Employed	18 (69)	15 (56)	
Retired	6 (23)	6 (22)	
Unemployed	2 (8)	6 (22)	
**Smoking history**, n (%)			0.21
Current smoker	4 (15)	9 (33)	
Ex-smoker	9 (35)	10 (37)	
Never smoker	13 (50)	8 (30)	
Comorbid medical conditions, n (%)	9 (35)	10 (37)	0.85
Comorbid behavioral disorder, n (%)	19 (73)	17 (63)	0.43
Epworth Sleepiness Scale	11.4 ± 3.8	12.2 ± 4.4	0.47
Insomnia Severity Index	18.2 ± 4.3	18.7 ± 4.1	0.63
PSQI	13.0 ± 3.4	13.7 ± 2.7	0.39
BDI-II	15.7 ± 8.5	16.1 ± 13.1	0.85
PCL	34.5±11.5	30.1±12.8	0.23
**Overnight polysomnography**			
Total sleep time (min)	324.7 ± 130.3	323.1 ± 93.8	0.95
Sleep onset latency (min)	26.7 ± 20.1	31.0 ± 24.2	0.48
Sleep efficiency (%)	74.7 ± 23.7	74.7 ± 22.1	0.98
N1 (%)	10.7 ± 6.4	7.7 ± 4.9	0.44
N2 (%)	64.5 ± 11.4	67.7 ± 14.1	0.39
N3 (%)	13.5 ± 11.4	10.2 ± 9.2	0.44
REM (%)	15.1 ± 9.4	14.1 ± 9.7	0.92
Apnea-hypopnea index, /h	29.2 ± 22.9	32.7 ± 25.3	0.65
Arousal Index, /h	8.2 ± 9.9	7.2 ± 9.6	0.87
Nadir SaO_2_, %	87.3 ± 3.6	85.9 ± 4.1	0.38
**Actigraphy**			
TIB (min)	513.2±78.1	502.2±67.8	0.62
TST (min)	369.7±64.1	364.3±55.5	0.77
SOL (min)	37.68±18.71	36.9±20.78	0.81
WASO (min)	43.0±19.3	49.8±24.8	0.11
SE (%)	72.4±9.2	73.1±10.7	0.80

ESZ = eszopiclone, BMI = body mass index, BDI-II = Beck Depression Inventory-II, PSQI = Pittsburgh Sleep Quality index, SE = sleep efficiency, SOL = sleep onset latency, TIB = time in bed, TST = total sleep time, WASO = wake after sleep onset

### Primary Outcome Analysis

The results of mixed-effects models for PSQI are summarized in Table 2. No significant effects were found on the time × treatment arm interaction with the 2 vectors (*p* = 0.88 and *p* = 0.73).

**Table 2:** Mixed effect linear models for primary and secondary outcomes from baseline to week 24

	PSQI	ISI	ESS	BDI	PCL
Intercept	14.77 [10.04, 19.5], < 0.001	19.66 [13.89, 25.44], < 0.001	9.37 [2.94, 15.77], 0.004	19.0 [6.87–31.12], 0.002	34.95 [13.41–56.48], 0.001
BBTI plus Esz					
Vector 1	-4.87 [-6.21, -3.53], < 0.001	-7.78 [-10.01, -5.54], < 0.001	-3.56 [-5.49, -1.64], < 0.001	-5.49 [-8.11, -2.87], < 0.001	-9.56 [-13.96, -5.1], < 0.001
Vector 2	-0.38 [-1.73, 0.98], 0.59	-0.55 [-2.82, 1.71], 0.63	0.27 [-1.71, 2.25], 0.79	1.15 [-1.56, 3.87], 0.41	-1.06 [-5.52, 3.39], 0.63
BBTI					
Vector 1	-4.73 [-6.06, -3.39], < 0.001	-6.24 [-8.52, -3.95], < 0.001	-2.62 [-4.87, -0.3], 0.02	-4.1 [-6.8, -1.4], 0.002	-4.65 [-9.12, -0.17], 0.04
Vector 2	-0.71 [-2.06, 0.64], 0.3	-2.39 [-4.74, -0.04], 0.04	-0.85 [-3.16, 1.45], 0.47	1.04 [-1.71, 3.82], 0.46	0.18 [-4.31, 4.67], 0.94
Group	0.75 [-1.08, 2.59], 0.43	0.56 [-1.98, 3.12], 0.67	0.82 [-1.64, 3.28], 0.51	0.44 [-4.02, 4.91], 0.85	-4.34 [-12.26, 3.56], 0.28
Vector 1 x Group	0.14 [-1.74, 2.03], 0.88	1.53 [-1.66, 4.72], 0.35	0.95 [-2.01, 3.97], 0.53	1.39 [-2.36, 5.13], 0.46	4.91 [-1.36, 11.18], 0.13
Vector 2 x Group	-0.33 [-2.24, 1.58], 0.73	-1.83 [-5.09, 1.42], 0.27	-1.13 [-4.17, 1.90], 0.47	-0.10 [-3.98, 3.77], 0.96	1.25 [-5.08, 7.58], 0.70

Unstandardized coefficients [95% confidence intervals] and P values are presented. Vector 1: 6 weeks vs. baseline post- randomization; positive values indicate baseline values larger than 6 weeks values. Vector 2: 24 weeks vs. 6 weeks post-randomization; positive values indicate 6 weeks values larger than 24 weeks values. BBTI = Brief Behavioral Treatment for Insomnia, ISI = Insomnia Severity Index, ESS = Epworth Sleepiness Scale, PSQI = Pittsburgh Sleep Quality Index, BDI = Beck Depression Inventory. All mean values are adjusted for outcome value at baseline, age, sex, AHI, and psychiatric comorbidity covariates

LMM revealed a main effect overtime indicating a significant decrease in PSQI scores from baseline to week 6 post-randomization in both the BBTI plus ESZ group (-4.87 [95% CI, -6.21 to -3.53]; *p* < 0.001) and the BBTI-only group (-4.73 [95%CI, -6.06 to -3.39]; *p* < 0.001). For the period extending from week 6 to week 24, a small and nonsignificant decrease in PSQI scores was observed for the BBTI plus ESZ group (-0.38 [95%CI, -1.7 to 0.9]; *p* = 0.58) and for the BBTI-only group (-0.71 [95% CI, -2.06 to 0.64]; *p* = 0.31).

Adjusted means and standard deviations of PSQI by assessment point are depicted in Table 3. The estimated mean difference of PSQI scores at the 6-week follow-up visit was 0.92, 95% CI, -2.80, 0.97; *p* = 0.33) indicating that participants in the BBTI-only group had a similar response to those in the BBTI plus ESZ group. A similar treatment effect was also observed at the 24-week visit (-0.58, 95% CI, -2.86, 1.68; *p* = 0.61).

**Table 3:** Self-reported primary and secondary outcome measures between the two treatment groups

	BBTI plus ESZ (*N* = 26)			BBTI (*N* = 27)			Treatment difference (95% CI)				
	Baseline	6 weeks	24 weeks	Baseline	6 weeks	24 weeks	BBTI plus ESZ vs. BBTI at 6 weeks	P value	BBTI plus ESZ vs. BBTI at 24 weeks	P value	
PSQI	13.0±3.4	8.1 ± 3.2	7.8 ± 4.2	13.8±2.7	9.0 ± 3.3	8.3 ± 3.7	-0.92 (-2.8,0.97)	0.33	-0.58 (-2.86, 1.68)	0.61	
ISI	18.2±4.3	10.4 ± 5.3	9.9±5.8	18.7±4.1	12.7 ± 3.2	10.3 ± 5.1	-2.3 (-4.95, 0.28)	0.07	-0.42 (-3.59, 2.74)	0.79	
ESS	11.4±3.9	7.7 ± 4.3	8.1 ± 5.2	12.2±4.4	10.2 ± 8.6	8.6 ± 5.1	-2.45 (-5.73, 0.83)	0.13	-0.5 (-3.62, 2.62)	0.74	
BDI-II	15.7±8.5	10.5 ± 7.9	11.6 ± 8.4	16.1±7.5	11.3 ± 8.3	12.5 ± 8.2	-0.89 (-5.65, 3.87)	0.71	-1.05 (-6.1, 4.02)	0.67	
PCL	34.5±11.5	24.9 ± 13.4	23.1 ± 13.1	30.1±12.7	25.9 ± 13.2	26.1 ± 13.7	-1.04 (-9.06, 6.97)	0.79	-3.05 (-12.14, 6.05)	0.51	

Baseline and stratum adjusted means and standard deviations estimated from the linear mixed models by time point. BBTI = Brief Behavioral Treatment for Insomnia, ISI = Insomnia Severity Index, ESS = Epworth sleepiness scale, PSQI = Pittsburgh Sleep Quality Index, BDI-II = Beck Depression Inventory-II. All mean values are adjusted for outcome value at baseline, age, sex, AHI, and psychiatric comorbidity covariates

### Secondary Outcomes Analyses

Table 2 shows the estimated treatment effects of the secondary clinical outcomes at 6- and 24-week follow-up visits. There were no significant group allocation treatment effects x time on insomnia symptoms, ESS, depression, or PCL scores. LMM revealed a main effect of time on ISI (-7.78; 95% CI, -10.01, -5.54; *p* < 0.001), ESS (-3.56; 95% CI, -5.49, -1.64; *p* < 0.001), BDI-II (-5.49, 95% CI, -8.11, -2.87; *p* < 0.001), and PCL (-9.56; -13.96, -5.1; *p* < 0.001), indicating that participants in all groups reported improvements in insomnia symptoms, excessive daytime sleepiness, depression scores, and burden of PTSD from baseline to 24 weeks across both study arms. The improvement in the performance of these self-reported instruments was observed mainly between baseline and follow-up visits at week 6, suggesting that no additional treatment improvement in propensity of sleepiness, and severity of depression between 6- and 24-week visits. Only ISI scores showed further decline with BBTI-only during that time interval (-2.39, 95% CI, -4.74, -0.04; *p* = 0.04) reflecting further abatement of insomnia severity.

Comparisons between participants in BBTI plus ESZ and BBTI-only revealed a trend for higher reduction in insomnia symptoms for those who received eszopiclone with BBTI at the 6-week follow-up (-2.3; 95% CI, -4.95, 0.28; *p* = 0.07) but not at the 24-week follow-up (*p* = 0.79), indicating a transient improvement attributed to the hypnotic agent (Table 3). No other differences between BBTI plus ESZ and BBTI-only groups at 6- or 24-week follow-up were reported for ESS, BDI-II, or PCL.

### Insomnia Response and Remission Rates

**Fig. 2:**
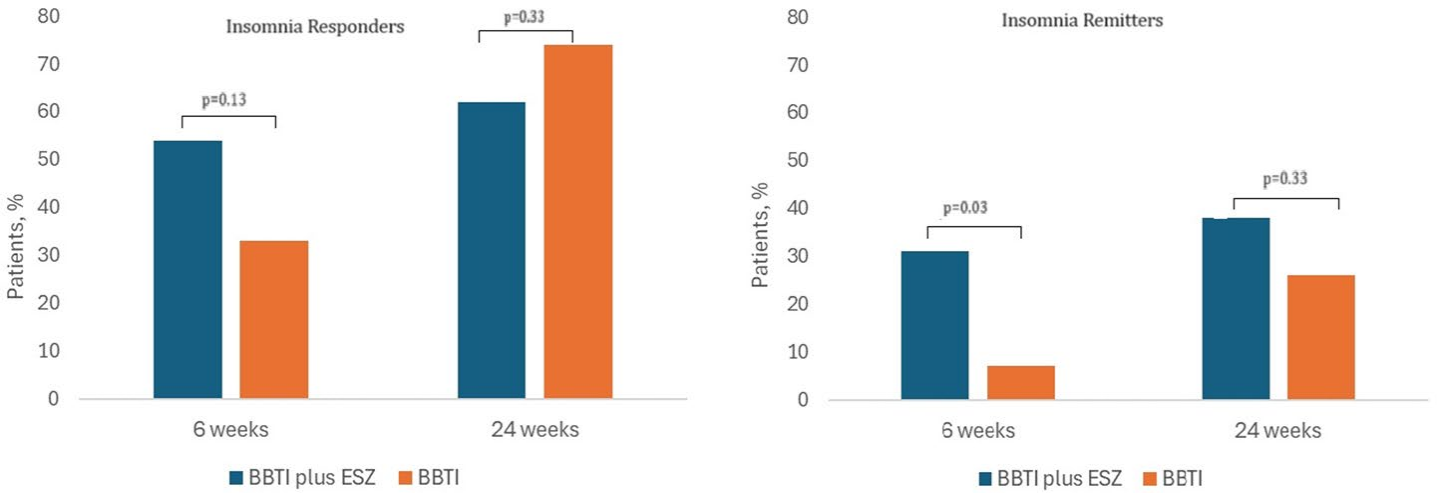
Percentage of insomnia responders and remitters after combination therapy of BBTI plus eszopiclone (COMB) or BBTI-only

The percentage of responders to insomnia treatment at 6- and 24-week assessment visit was no different between participants in the BBTI plus ESZ arm versus BBTI-only arm. Analytically, 14 of 26 participants in the BBTI plus ESZ met the ISI criteria for responders compared with 9 of 27 in the BBTI-only group, (54% versus 33%; *p* = 0.13) at the end of the 6-week treatment period. At 24 weeks, 16 out of 26 participants who received BBTI plus ESZ and 20 of the 27 who received BBTI-only treatment were characterized as responders (62% versus 74%; *p* = 0.33) (Fig. 2). In contrast, combination therapy produced a higher remission rate at the 6-week follow-up compared to BBTI-only but not at the 24-week endpoint. Of the 26 participants assigned to BBTI plus ESZ, 8 achieved the threshold of remission at 6-week follow-up compared to 2 out of 27 participants assigned to BBTI-only (31% versus 7%; *p* = 0.03). At the 24-week endpoint, 10 out of 26 participants achieved remission in the BBTI plus ESZ while 7 out of 27 in the BBTI-only were considered as remitters (38% versus 26%; *p* = 0.33).

### Actigraphy-Derived Sleep Indices

Results of the mixed-effects models for actigraphy parameters are summarized in Table S1 (Online Resource). After 6 weeks post-randomization, simple effects analyses revealed a significant increase in total sleep time (TST) (34.6 min, 95% CI [4.66–64.55]; *p* = 0.02) and a decrease in sleep onset latency (SOL) (-17.82 min, 95% CI [-25.23, -10.40]; *p* < 0.001) from baseline to the 6-week treatment period for the BBTI plus ESZ group. There was also a significant improvement in sleep efficiency (SE) in the BBTI plus ESZ group during the same period (9.76%, 95% CI [4.02, 15.50]; *p* = 0.001) but not for time spent in bed (*p* = 0.19) or WASO (*p* = 0.07). In contrast, there was a significant decrease in time in bed (TIB) (-33.0 min, 95% CI [-64.71, -1.26]; *p* = 0.04), SOL (-8.63 min, 95% CI [-16.59, -1.07]; *p* = 0.03), and in wake after sleep onset (WASO) (-7.49 min, 95% CI [-15.03, 0.02]; *p* = 0.03) in the BBTI-only group but not in TST (*p* = 0.24) or SE (*p* = 0.29). After the 24-week follow-up visit, there were no significant additional treatment effects for any of the groups pertaining to TIB, TST, WASO, or SE. A significant group x time interaction was observed for SOL during the 6-week treatment phase indicating that the BBTI plus ESZ group experienced a greater reduction in SOL compared with patients treated with BBTI-only (difference of 9.98 min, 95% CI [1.14, 19.72]; *p* = 0.03). However, this difference between the two treatment groups was not sustained at the 24-week follow-up (*p* = 0.31).

Table S2 (Online Resource) shows the adjusted means and standard deviations of sleep actigraphy for each treatment by week 6 and week 24. Examining treatment differences at each time point showed no significant change in TIB, TST, WASO, or SE at 6 or 24 weeks. Only those patients who were randomized to BBTI plus ESZ had a lower SOL at a 6-week follow-up visit compared to those assigned to BBTI-only (-8.21 min, 95% CI [-16.13, -0.29]; *p* = 0.04).

### CPAP Use

Results of the LMM for CPAP use revealed no group x time interaction effects. During the first 6 weeks of treatment, significant increases in utilization of CPAP from baseline were observed in the BBTI plus ESZ group (118.50 min; 95% CI, 72.38, 164.62; *p* < 0.001) but not in the BBTI-only group (30.56 min; 95% CI, -14.70, 75.82; *p* = 0.19) (Table 4). Additionally, patients who received BBTI plus ESZ had used on average CPAP 75.8 min more than those who were assigned to BBTI-only (difference 75.8 min; 95% CI, 17.18, 134.72; *p* = 0.01). No additional improvement was recorded between the 6-week and the 24-week follow-up visits in either the BBTI plus ESZ arm (-21.23 min, 95% CI, -70.39, 24.89; *p* = 0.37) or the BBTI-only arm (26.15 min, 95% CI, -19.11, 71.41; *p* = 0.26). However, CPAP utilization increased significantly between baseline and the 24-week follow-up in both the BBTI plus ESZ arm (97.27 min, 95% CI, 51.15, 143.39; *p* < 0.001) and the BBTI-only arm (56.71 min, 95% CI, 11.44, 101.96; *p* = 0.01). Although the percentage of nights with CPAP device use exceeding 4 h exhibited a trend toward higher CPAP use in favor of the BBTI plus ESZ arm at the 6-week follow-up, there were no significant differences in CPAP adherence between the two groups at either the 6-week or the 24-week follow-up visits (BBTI plus ESZ 38% and 35% versus BBTI-only 15% and 19%, respectively) (*p* = 0.06 and *p* = 0.22, respectively).

**Table 4:** CPAP usage for each treatment group

CPAP usage (min)	Baseline	Week 6	Week 24
BBTI plus ESZ	73.23±88.01	191.73±112.67	170.5±153.24^†^
BBTI	85.22±72.51	115.78±100.29	141.92±92.25*

CPAP = continuous positive airway pressure, BBTI = Brief Behavioral Treatment for insomnia, ESZ = eszopiclone

Main effect of time in each group indicated in ‘week 24’ column: *p < 0.05; ^†^*p* < 0.001

### Adverse Events

A total of 12 adverse events were registered during the clinical trial. These events occurred in 6 (11%) patients. Four were randomized to BBTI plus ESZ and 2 to BBTI-only. Adverse events were not significantly different between the BBTI plus ESZ and the BBTI-only group (Fisher’s exact test *p* = 0.20). The most reported adverse events were headache (*n* = 5) and unpleasant taste (*n* = 3). Other adverse events included transient dizziness, nasal congestion, and loss of appetite. None of these events were categorized as serious and these issues were resolved prior to or by the end of the 6-week follow-up.

## Discussion

The findings of this pilot RCT suggest that while both BBTI-only and BBTI plus ESZ were associated with significant improvement in sleep quality over the initial 6-week treatment phase in PTSD patients with COMISA, combining BBTI with a short course of ESZ did not confer additional long-term benefit over BBTI-only. Parallel observations were recorded for excessive daytime sleepiness and depression. The study showed also a higher remission rate of insomnia in the short term with combination therapy compared to BBTI-only although both interventions yielded comparable rates over time. Equally, CPAP use increased in both treatment groups, with a greater rise observed in those receiving BBTI plus ESZ. However, this difference was primarily evident during the initial treatment phase and may not necessarily reflect long-term adherence patterns. However, the addition of ESZ to BBTI did not lead to sustained long-term improvements in CPAP utilization or adherence compared to BBTI-only.

Despite the proven efficacy of CBT-I in the management of insomnia [14, 43, 44], a combined behavioral and pharmacological approach has been considered an alternative approach for patients who are less likely to respond to psychotherapy alone [26]. This trial is the first to extend this modality to COMISA patients who exhibit daytime symptoms of insomnia and poor CPAP usage. Our results suggest that combining BBTI with pharmacotherapy did not impart significant long term benefit in sleep quality of life, propensity for sleep, depression severity, or burden of PTSD despite a trend toward a more rapid onset of improvement in objective sleep parameters. Earlier studies combining CBT with hypnotics have similarly demonstrated either no additional benefit or slight advantage over monotherapy in targeting chronic insomnia [22, 25]. This discrepancy has been the subject of long debates. Objective markers that denote “good sleep” do not necessarily overlap with patients’ self-reported ratings of sleep quality [45–47]. As a matter of fact, higher sleep quality was better correlated with self-report measures of anxiety and depression than objective sleep indices [48]. This poor agreement between objective sleep methods and self-reported sleep quality indicates a need for more basic investigations on linking perception of “good” sleep experiences with sleep neurophysiology.

We found that the addition of eszopiclone to BBTI conferred a higher remission rate of insomnia than BBTI alone after the 6-week treatment course. This observation suggests that eszopiclone might have enhanced compliance with BBTI. It should be noted however that the remission rates in our patients with COMISA (7% for BBTI at 6 weeks) were significantly lower than the one reported by Morin and colleagues [20] in patients with chronic insomnia (39% for CBT-I for the same period). We surmise that the discrepancy in rates of remission between the two studies could be attributed to the presence of comorbid PTSD in our study population. Patients with PTSD often experience persistent insomnia symptoms that are often more resistant to standard treatments [49]. Hyperarousal, intrusive traumatic memories, and recurrent nightmares often lead to maladaptive associations by pairing sleep-related cues with wakefulness and arousal [50]. 

With regard to CPAP use, recent trials examining the effect of psychotherapy on CPAP adherence in COMISA patients were mixed [16–18, 44]. In this study, our findings suggest that the addition of eszopiclone to BBTI may have facilitated CPAP usage and contributed to a greater nightly CPAP use during the initial period of psychopharmacotherapy compared to BBTI alone. However, further research is needed to confirm these effects and determine their long-term clinical significance. A presumed reason for the improved adherence with nights of CPAP use is the reduced sleep onset latency with combination therapy which has the potential to reduce awakenings associated with the CPAP-interface. Prior studies have implicated eszopiclone with better adherence to CPAP [51, 52]. While the addition of eszopiclone showed initial benefits in increasing CPAP use, the waning of CPAP adherence after withdrawal of eszopiclone and the gradual improvement in CPAP utilization with BBTI-only abrogated the early advantage of combination therapy. However, when comparing baseline to 24-week follow-up visits, both groups demonstrated significant increases in CPAP utilization, indicating that the initial improvements were generally maintained over the long term.

Several contextual limitations may have influenced the results of the trial. First, the study comprised older male veterans with PTSD limiting the generalizability to women and non-veterans without PTSD. Second, the lack of eszopiclone blinding may have biased outcome estimates. Participants in non-blinded clinical trials may report symptoms differently from blinded ones because of response bias [53]. Third, the prediction of positive treatment responses may be embedded in insomnia phenotypes. Recent investigations have shown that sleep onset insomnia and early morning awakenings were linked to poor CPAP adherence [10, 54]. Others found sleep maintenance symptoms to be more prominent [12]. Because of our relatively small sample size, we did not examine these associations for lack of statistical power. Fourth, the combination therapy involved eszopiclone, a non-benzodiazepine γ-aminobutyric acid receptor agonist, along with BBTI. We cannot ascertain whether the findings of this trial can be replicated with other classes of hypnotics. Fifth, although we considered including a placebo in the BBTI-only arm to match the eszopiclone use, we elected against it because our research aim was not about documenting treatment efficacy relative to placebo but the comparative effectiveness of BBTI plus ESZ versus BBTI-only. Similarly, we have not included a CPAP-only control because many participants on the waitlist may drop out before receiving treatment for insomnia [55, 56].

## Conclusions

In veterans with PTSD, adding eszopiclone to BBTI did not confer long-term benefit in improved sleep quality compared to BBTI-only in the management of COMISA. When initiated with cognitive behavioral therapy, the addition of eszopiclone for a limited time is an effective and safe regimen to expedite remission from insomnia symptoms and to improve CPAP utilization. Future studies aiming at determining optimal therapy based on insomnia phenotypes are warranted.

## Declarations

### Disclaimer

The views expressed in this article are solely those of the authors and do not reflect an endorsement by or the official policy of the U.S. Army, the Department of Defense (DoD), the Department of Veterans Affairs (VA), or the U.S. Government. This material is the result of work supported with resources and the use of facilities at the VA Western New York Health Care System.
